# Novel Software for High-level Virological Testing: Self-Designed Immersive Virtual Reality Training Approach

**DOI:** 10.2196/44538

**Published:** 2023-06-21

**Authors:** Huey-Pin Tsai, Che-Wei Lin, Ying-Jun Lin, Chun-Sheng Yeh, Yan-Shen Shan

**Affiliations:** 1 Department of Pathology, National Cheng Kung University Hospital College of Medicine National Cheng Kung University Tainan Taiwan; 2 Department of Medical Laboratory Science and Biotechnology College of Medicine National Cheng Kung University Tainan Taiwan; 3 Medical Device Innovation Center National Cheng Kung University Tainan Taiwan; 4 Department of Biomedical Engineering College of Engineering National Cheng Kung University Tainan Taiwan; 5 Institute of Clinical Medicine College of Medicine National Cheng Kung University Tainan Taiwan; 6 Division of General Surgery, Department of Surgery, National Cheng Kung University Hospital College of Medicine National Cheng Kung University Tainan Taiwan

**Keywords:** design, immersive, virtual reality, VR, high-level clinical virology, skill training, testing, virology, virological, medical education, clinical practice, simulation, biotechnology, molecular, detection, pathogen, development, software, teaching

## Abstract

**Background:**

To ensure the timely diagnosis of emerging infectious diseases, high-tech molecular biotechnology is often used to detect pathogens and has gradually become the gold standard for virological testing. However, beginners and students are often unable to practice their skills due to the higher costs associated with high-level virological testing, the increasing complexity of the equipment, and the limited number of specimens from patients. Therefore, a new training program is necessary to increase training and reduce the risk of test failure.

**Objective:**

The aim of the study is to (1) develop and implement a virtual reality (VR) software for simulated and interactive high-level virological testing that can be applied in clinical practice and skills building or training settings and (2) evaluate the VR simulation’s effectiveness on reaction, learning, and behavior of the students (trainees).

**Methods:**

Viral nucleic acid tests on a BD MAX instrument were selected for our VR project because it is a high-tech automatic detection system. There was cooperation between teachers of medical technology and biomedical engineering. Medical technology teachers were responsible for designing the lesson plan, and the biomedical engineering personnel developed the VR software. We designed a novel VR teaching software to simulate cognitive learning via various procedure scenarios and interactive models. The VR software contains 2D VR “cognitive test and learning” lessons and 3D VR “practical skills training” lessons. We evaluated students’ learning effectiveness pre- and posttraining and then recorded their behavior patterns when answering questions, performing repeated exercises, and engaging in clinical practice.

**Results:**

The results showed that the use of the VR software met participants’ needs and enhanced their interest in learning. The average posttraining scores of participants exposed to 2D and 3D VR training were significantly higher than participants who were exposed solely to traditional demonstration teaching (*P*<.001). Behavioral assessments of students pre- and posttraining showed that students exposed to VR-based training to acquire relevant knowledge of advanced virological testing exhibited significantly improved knowledge of specific items posttraining (*P*<.01). A higher participant score led to fewer attempts when responding to each item in a matching task. Thus, VR can enhance students’ understanding of difficult topics.

**Conclusions:**

The VR program designed for this study can reduce the costs associated with virological testing training, thus, increasing their accessibility for students and beginners. It can also reduce the risk of viral infections particularly during disease outbreaks (eg, the COVID-19 pandemic) and also enhance students’ learning motivation to strengthen their practical skills.

## Introduction

Several outbreaks of infectious viral diseases with high fatality rates have occurred over the last 20 years: COVID-19 (caused by the SARS-CoV-2 virus), the new avian influenza virus (ie, H7N9), and the respiratory syncytial virus. These have all caused high hospitalization rates [1-4]. Immunocompromised patients are vulnerable to severe diseases caused by cytomegalovirus (CMV), herpes simplex virus (HSV), and herpes zoster virus infections [5-8]. Thus, a timely diagnosis is necessary for clinicians to determine the optimal treatment plan. Accordingly, rapid diagnoses with high fidelity comprise an important requirement for clinical virology laboratories.

The use of high-tech molecular technology for the rapid detection of pathogens in clinical specimens has gradually become the gold standard for clinical virological testing [9,10]. Furthermore, quantitative or qualitative test analysis is used as an important indicator for medical diagnosis, treatment tracking, risk assessment, and health care information.

Molecular techniques are an important tool for clinical testing; however, the cost of molecular test reagents is high. Further, special and repetitive training is required to operate the testing equipment; consequently, clinical virology laboratories frequently prevent medical technology (MT) interns from practicing these procedures on their equipment. There are similar problems in teaching in other specialized areas involving clinical medical skills. For example, surgical training has always relied heavily on practical operations to ensure that physicians acquire the necessary technical proficiency to perform surgical procedures. After observing surgical procedures and animal experiments demonstrated by the teacher, intern surgeons practice the relevant technical skills while being supervised by the teacher. This teaching method may be limited by various factors, including medical ethics, working hours, and medical disputes. Therefore, scholars in the field of surgical teaching have recently developed simulation tools to teach residents to perform surgery. Virtual reality (VR) or augmented reality or tactile computer programs can simulate the surgical environment for teaching purposes and apply minimally invasive surgical robots [11,12].

Traditional teaching methods transmit medical knowledge via lectures and textbooks. However, many teachers have recently begun to introduce 2-way communication learning methods such as problem-based learning and flipped education. These methods aim to replace the prevalent unidirectional cramming teaching method by transforming students from passive to active agents; this can improve their learning effectiveness [13-17]. Although each specialized field has its own special required skills and methods, clinical practice courses play a pivotal role in medical technologists’ development. Students must hone their practical skills to reduce their chance of failure in real-world settings. Therefore, the depth and breadth of students’ clinical training and whether they can actually engage in operational exercises will affect their learning.

The fully automatized BD MAX system developed by Beckton Dickinson Company integrates 2 functions: fluorescent polymerase chain reaction (PCR) detection and nucleic acid extraction. This detection system has 24 independent PCR heating elements, 5 different fluorescence channels, and can run different programs simultaneously. It processes 96-120 samples in 8 hours. It also has high efficiency for the detection of pathogens.

The BD MAX system is used in modern clinical virology laboratories for the detection of multiple clinical microbial nucleic acids present in emergent infectious pathogens. Although the BD MAX is powerful, the cost of reagents is high. Thus, clinical virology laboratories often prevent MT interns from practicing with their equipment. Schools often have scarce funds to obtain such advanced and expensive equipment for students to learn. Further, even if the students manage to understand the principles of the procedure after analyzing the operation manual and observing the workflow of a well-trained medical technologist, they often cannot access appropriate practical training. Therefore, it is critical to develop new teaching methods that allow MT students to put their skills into practice so that those who do not have the opportunity to engage in real-world practice with this specialized technology can still receive training.

VR comprises a virtual environment that is similar to reality. Computer technology can simulate a high-fidelity space and allow users to immerse themselves inside that environment. In a VR environment, users wear a special display device (ie, VR headsets) to enter the simulation. In this space, the operator can use a controller or keyboard to interact with the virtual environment. Importantly, VR does not aim to replace real space. By combining a camera’s recognition technology and a computer program, a set picture appears in the lens, and the corresponding virtual objects appear. However, high-tech VR products have not yet been developed and used in the field of skill training for virological testing. Thus, this study designed and implemented a VR software to train MT students to use a high-level virological testing system (ie, BD MAX) and evaluated students’ learning effectiveness.

## Methods

### VR System Description

The proposed study was developed based on Oculus Rift and cross-platform Unity. Oculus Rift is a VR goggle developed and manufactured by Oculus VR. The Rift has 2 PenTile organic light–emitting diode displays and offers 1080×1200 resolution per eye, a 90-Hz refresh rate, and a 110-degree field of view. The device also features rotational and positional tracking as well as integrated headphones that provide a 3D audio effect. The separation of the lenses is adjustable using a slider on the bottom of the device to accommodate a wide range of interpupillary distances. The Rift allows for full 6-degrees-of-freedom rotational and positional tracking. Tracking is performed by Oculus’s Con.

### Creating Course Features

We designed several virtual clinical and experimental scenarios for students to develop the relevant skills for virological testing in a realistic setting. By using VR, students can develop practical skills by engaging with interactive content, without being limited to merely reading about standard operating procedures.

### Study Population

Senior-year students from the Department of Medical Laboratory Science and Biotechnology (ie, MT students) at the College of Medicine of National Cheng Kung University (NCKU) in Tainan, Taiwan enrolled in the Clinical Virology Internship Course. Of these, 31 MT students participated in a 2D VR cognitive test and a 3D VR practical simulation.

### Cooperation Between the MT and Biomedical Engineering Groups in the Study

The teachers of the MT department at NCKU were responsible for designing the lesson plan and questionnaires, as well as evaluating students’ learning effectiveness. The department of biomedical engineering (BME) was responsible for the development of the VR software. The VR-based training was carried out in the VR classroom of the BME department. System operation was carried out in the clinical virology room of the Pathology Department at NCKU Hospital.

### The Cross-Disciplinary MT and BME Team

Since March 2019, we have discussed the subject of VR-based teaching plans as well as clinical virology teachers’ needs and feedback on the VR software described in this study. The BME group adjusted and optimized the VR software for high-level clinical virological testing after regular brainstorming and discussion. As of June 2022, there have been a total of 45 meetings.

### Design of the High-Level Clinical Virological Testing Lessons

The BD MAX instrument is an automatic virus nucleic acid detection system and was selected as the basis for our VR project. It is currently used to detect a variety of microorganisms (eg, HSV, varicella-zoster virus [VZV], and cytomegalovirus) at the NCKU Hospital’s clinical virology laboratory.

#### 2D VR “Cognitive Test and Learning” Lessons

**Figure 1:**
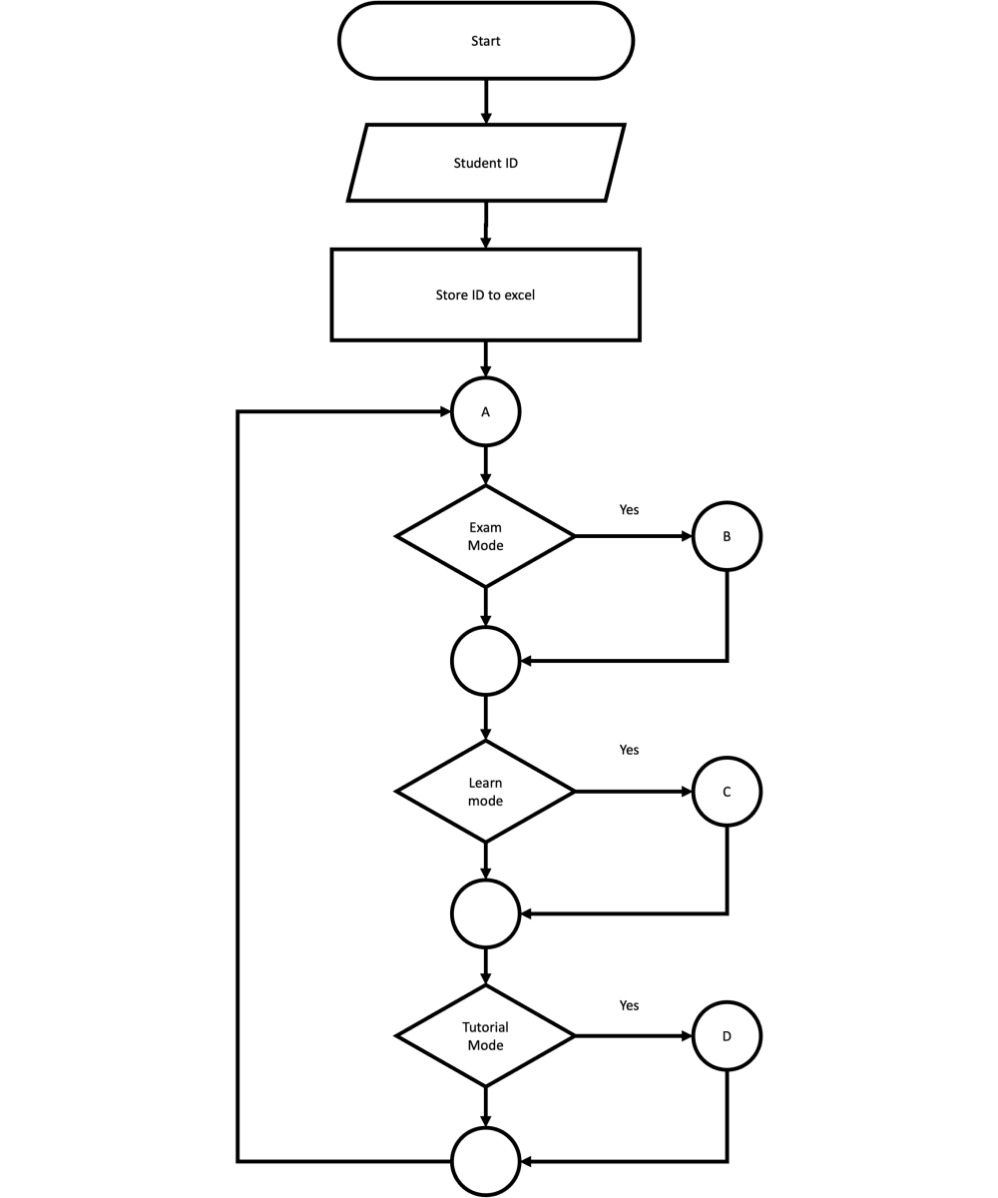
User activity diagram of the proposed research.

**Figure 2:**
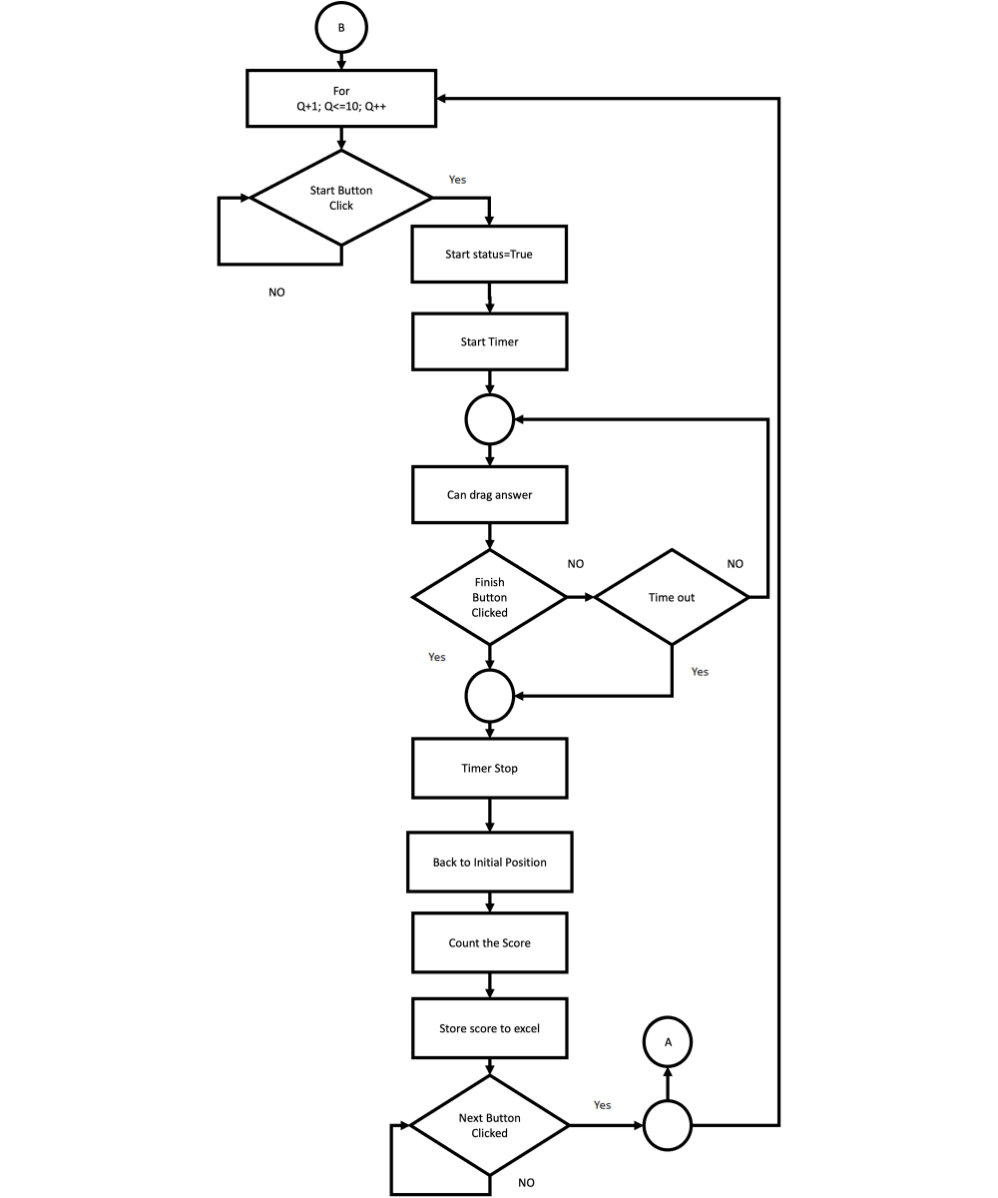
Flowchart for exam mode.

**Figure 3:**
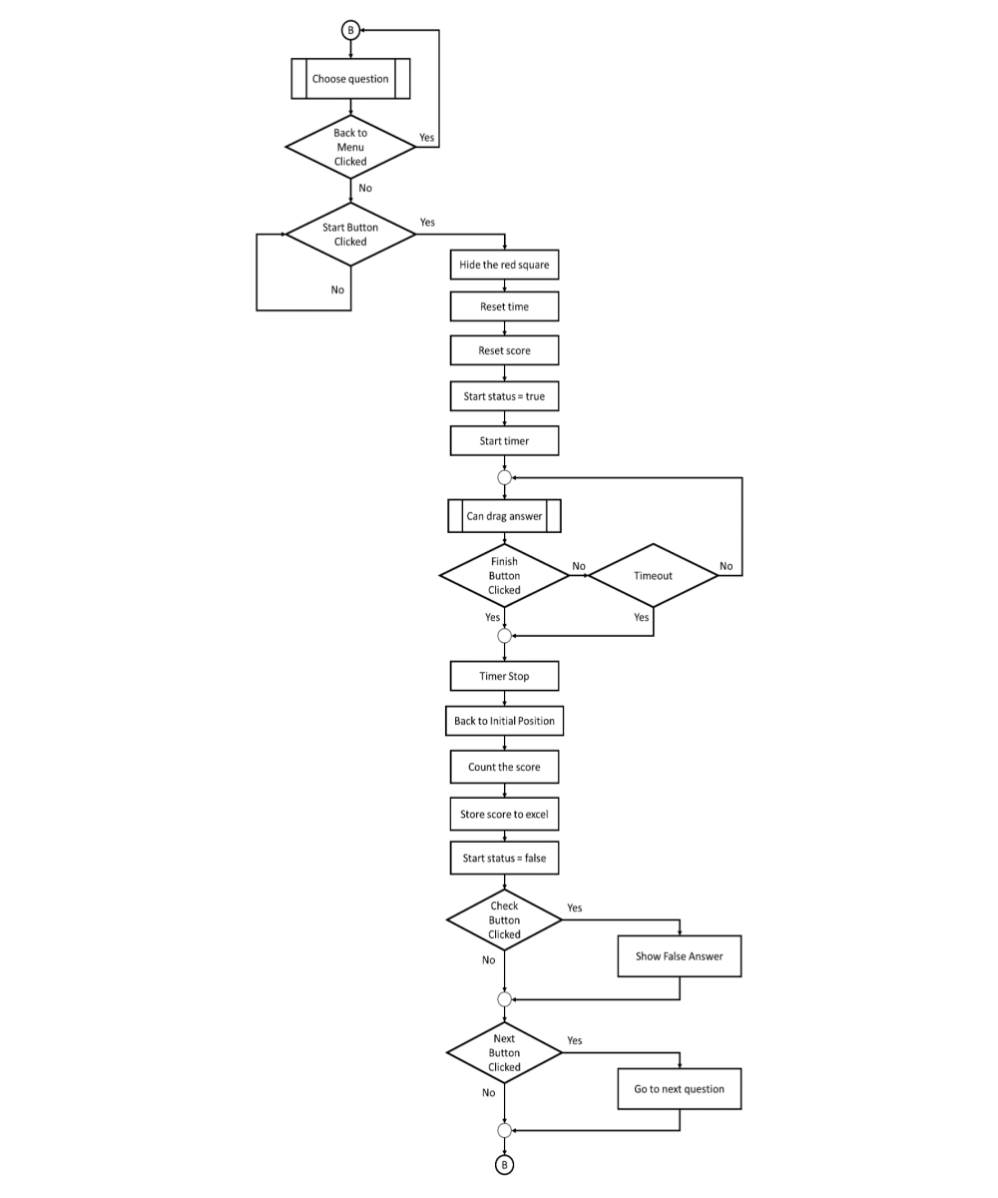
Flowchart for learn mode.

**Figure 4:**
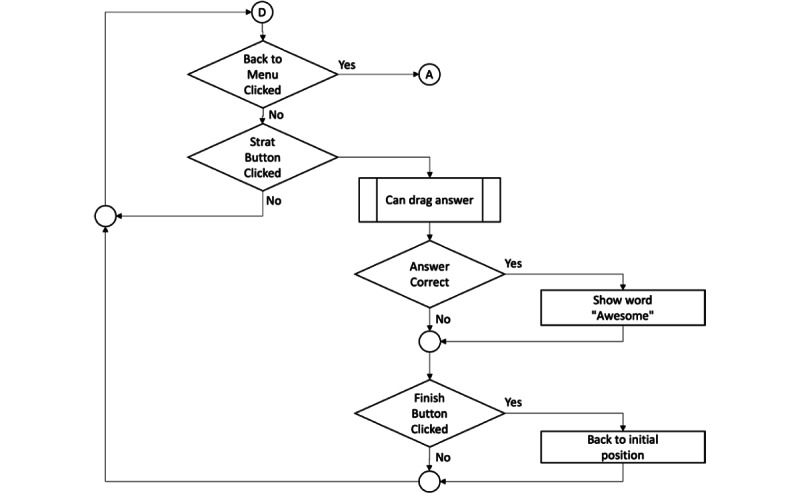
Flowchart for tutorial mode.

**Figure 5:**
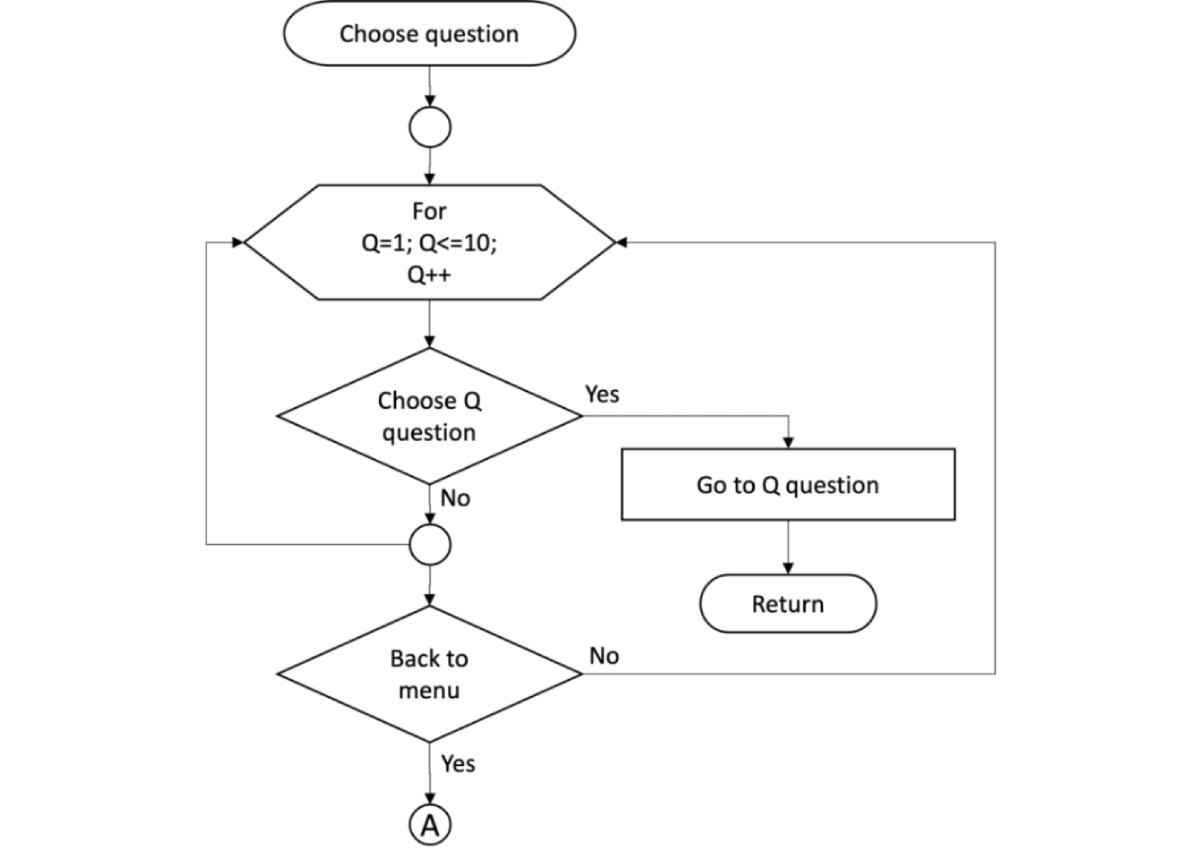
Subflowchart for choose question.

**Figure 6:**
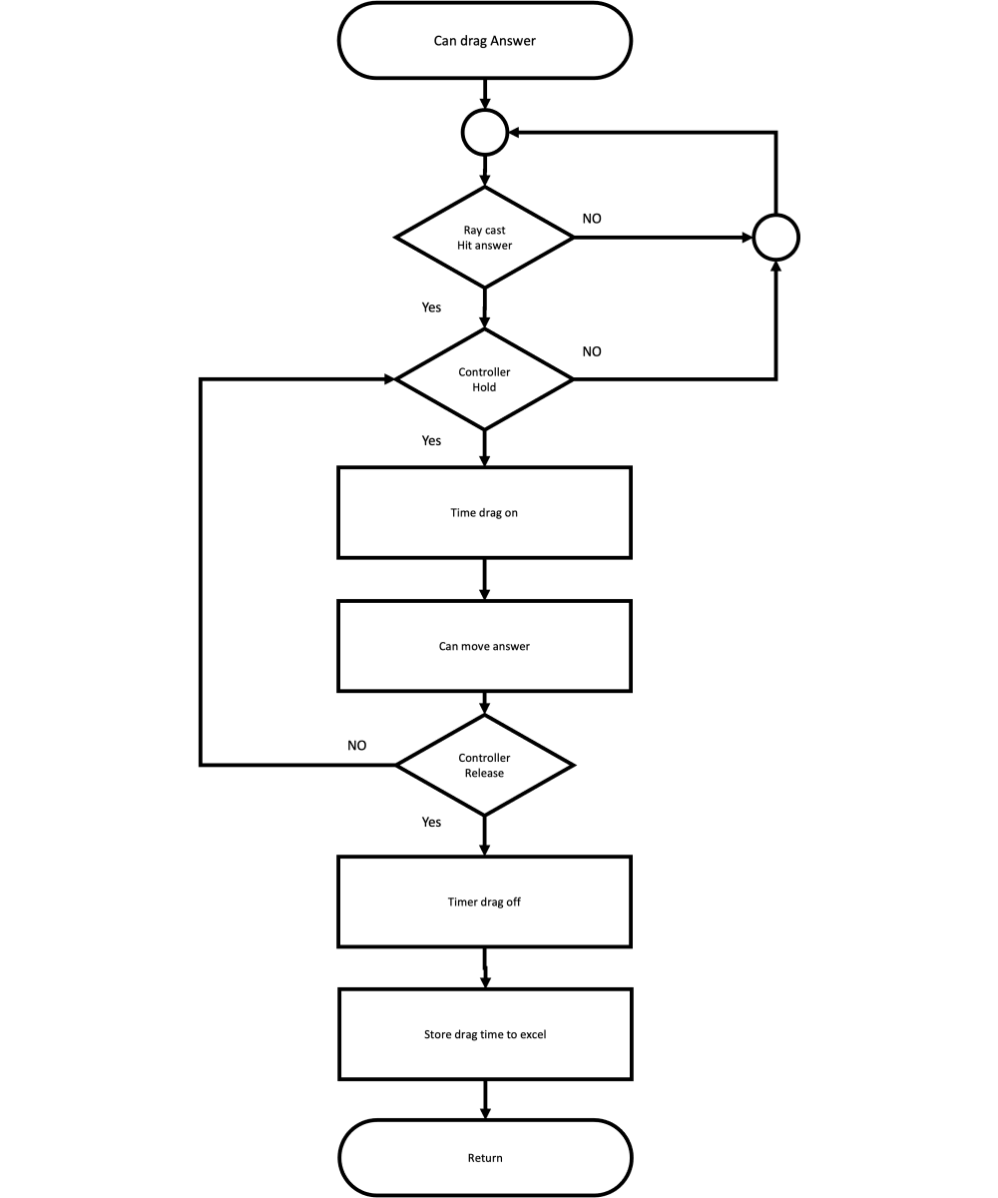
Subflowchart for drag answer.

**Figure 7:**
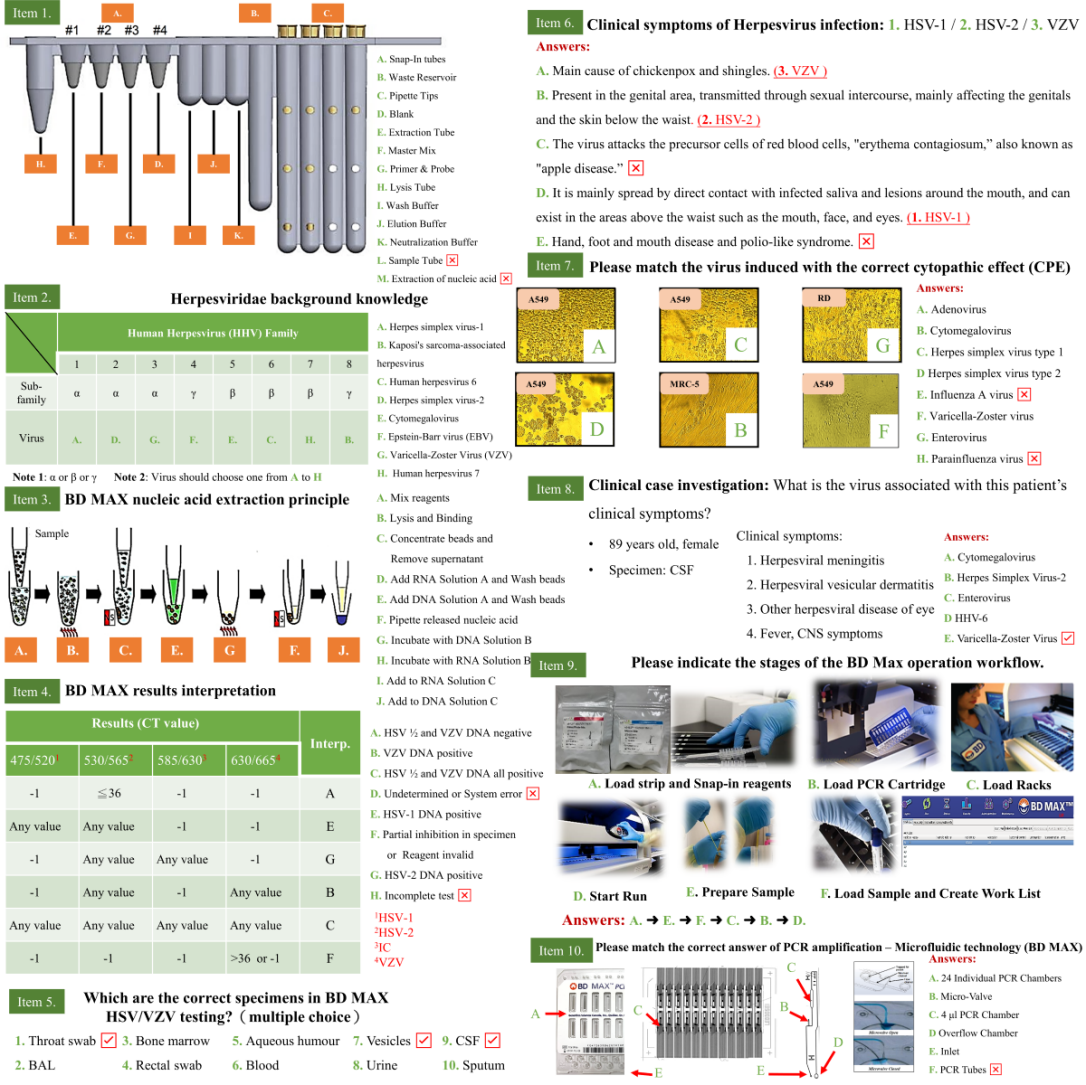
Ten designed items on the usage of BD MAX for 2D VR lessons. BAL: bronchoalveolar lavage; CNS: central nervous system; CSF: cerebrospinal fluid; EBV: Epstein-Barr virus; HHV: human herpesvirus; PCR: polymerase chain reaction; VR: virtual reality; VZV: varicella-zoster virus.

**Figure 8:**
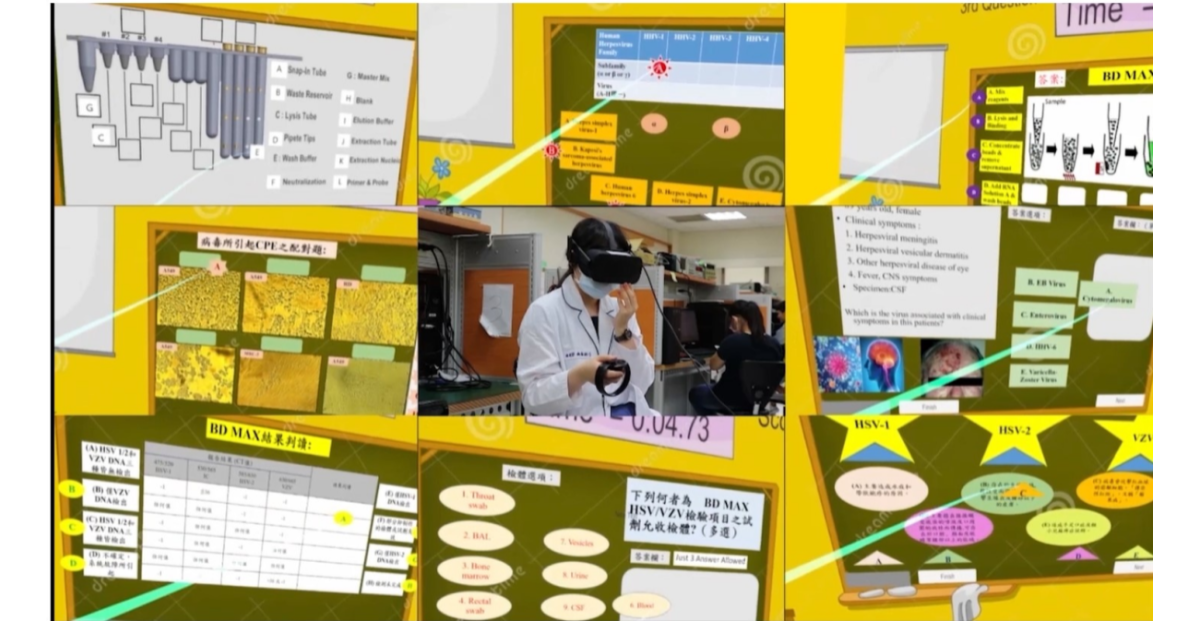
The picture of a 2D VR scenario. VR: virtual reality.

The self-developed VR teaching software had both a test mode and a learning mode. The computer software registered students’ score and immediately provided them with detailed feedback on the results. Combined with the test items (which assessed students’ knowledge regarding HSV and VZV PCR), a series of matching tasks was designed to prepare learners to interact with the VR environment. The difficulty level of the items was measured by the frequency of drag-and-drops (the term “drag” refers to the behavior of sliding the button to the answer before actually selecting the answer in the VR scenario) when students attempted to answer a given item. There were 10 items designed on the usage of BD MAX. The user activity diagram of the proposed research is shown in Figure 1, and the detailed flowchart of each mode is shown in Figures 2-6. The user manual is shown in Multimedia Appendix 1. Items 1-10 are shown in Figure 7, and the picture of VR scenario is shown in Figure 8.

- Test mode: Students had to answer the items in order; the system automatically calculated the score after the allotted time ended. Relaxing music played in the period between items to avoid visual fatigue affecting the score.
- Learning mode: Students were allowed to complete the tasks at their own pace. There was no limit on the time or number of attempts, and they could receive feedback on the results in real time.

#### 3D VR “Practical Skills Training” Lesson

**Figure 9:**
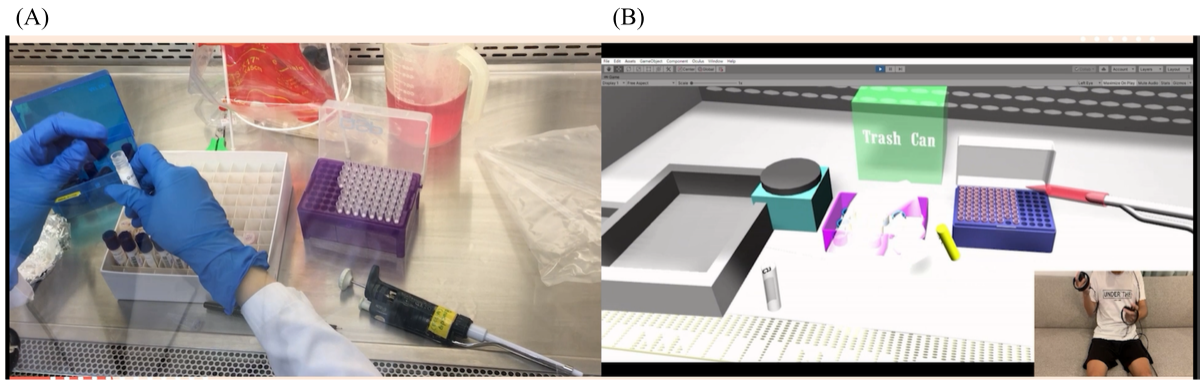
(A) Real scenario of operating the clinical specimen by medical technologist and (B) 3D virtual reality scenario.

**Figure 10:**
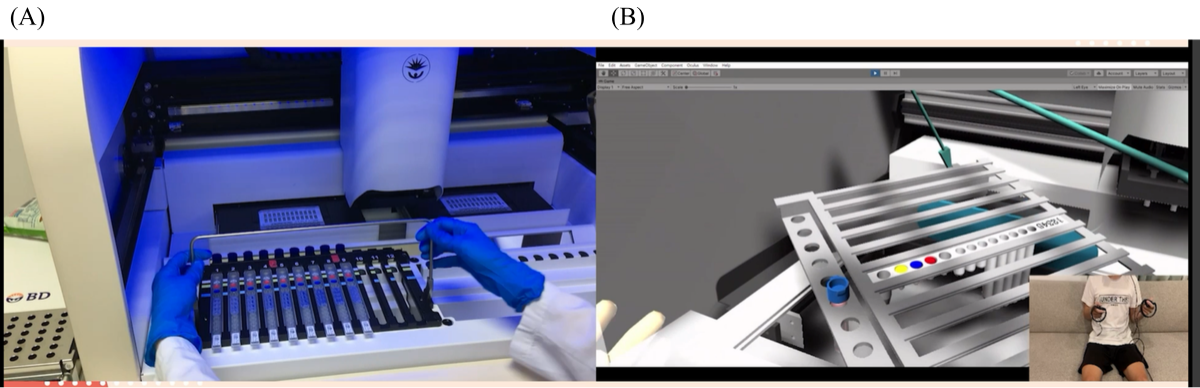
(A) Real scenario of operating BD MAX instrument by a medical technologist and (B) 3D virtual reality scenario.

The virology (MT) group provided a video of the actual operation process using the BD MAX system (while detailing each used component) to the BME team to create a high-fidelity 3D VR component. We recorded the virological testing process or an introduction to the structure of the automatic testing system. We also used computer software to divide the procedure’s steps on BD MAX and performed the procedures. We disassembled the steps of the practical procedure, and the design internship students could control the 3D objects to simulate the experimental process through the human-machine interface. The learners performed the entire practice process in a virtual learning setting. In the VR environment, there was a setting to prompt the learner to proceed to the next step so they could simulate the actual testing procedure. The interactive mode included using the hands in the VR simulation to place the objects in the correct position: a special color was displayed if the order was correct. A warning was displayed if the order or position was wrong—students were instructed to try again until they were correct. Real scenarios are shown in Figure 9A and Figure 10A. 3D VR scenarios are shown in Figure 9B and Figure 10B.

#### Creation of the VR Content

- The research team has Unity 3D software programming skills. The Unity 3D VR engine can operate VR scripts using the programming languages C# or JavaScript to thus control the VR objects.
- Art production and 3D object production: After establishing the teaching script, the research team developed high-fidelity 3D VR components by referencing interactive or experimental components from the teaching script.
- Sensor-based and user interface programming: To achieve high interactivity between the user and the VR learning content, we used the sensors inside the VR headset to register the user's movements. This research project used the Oculus Rift as a VR teaching device. The sensors in the Oculus Rift include infrared sensors (that track the user's body movements) and joysticks (that register the user's button presses). The research team programmed scripts to grasp the user's movements through the sensor and used it to drive the system’s interface. An interactive mode tracked the user's behavior to control 3D objects and the process of learning or experimenting through the human-machine interface.
- 3D object manipulation and interactive programming: The 3D object manipulation and interactive program was built into the VR scenario. The project achieved the VR teaching purpose when the user controlled the 3D objects to process learning or experimentation through the human-machine interface.
- System architecture and human-machine interaction
  Step 1. The user grasps the sensors placed on both sides of the computer and places them in their palms.
  Step 2. The user operates the VR system using the joystick.
  Step 3. The computer receives the operation command from the joystick and the sensor data and then displays it on the screen.
  Step 4. The VR helmet provides simulation scenarios to the user.

After all the virtual scenarios were tested, the VR classroom provided learners with a virtual space for learning and interaction.

#### Introduction of the VR Software and Interactive Model Into Clinical Practice Teaching and Skill Training

Our research team used the concept of software reliability to test the VR software’s stability. First, we developed a test plan to analyze each functional requirement, the expected trigger function, and whether the results were correct. The failure status of the software was defined according to 3 levels (high, medium, and low). Reliability coverage testing was subsequently performed. Coverage tests were conducted for input domain behavior, input variables, and their combinations, as well as various usage functions. After the tests were completed, we wrote a test report based on the failure condition and its definition and then made the necessary corrections. Medium- and high-level risks were completely resolved before the VR software was presented to users for testing. The users were finally allowed to use the VR software.

### Ethics Approval

The Institutional Review Board of NCKU Hospital approved the study (B-ER-109-432). All of the students were informed about the aim and protocol of the study, and written informed consent was obtained from all of them.

### Learning Effectiveness Evaluation

The levels of the Kirkpatrick model were used to investigate reaction, learning, and behavior of the students (trainees). We designed questionnaires of VR application learning effectiveness evaluation for participants to fill out anonymously, including the “satisfaction of using different learning materials” (reaction level) and the “self-assessment questionnaire of participants’ knowledge application” (behavior level). The score of the item in the questionnaires was as follows: very disagree got 1 point, disagree got 2 points, no comment got 3 points, agree got 4 points, and very agree got 5 points. Questionnaires of behavior level were conducted during pre- and posttraining. We also used 10 items of “cognitive test and learning” lessons (Figure 7) for written tests with registered identification number to evaluate learning level of students’ background knowledge of HSV or VZV and the operations of BD MAX. The score of each item was as follows: 11 points in item 1, 16 points in item 2, 7 points in item 3, 6 points in item 4, 3 points in item 5, 3 points in item 6, 6 points in item 7, 1 point in item 8, 6 points in item 9, and 6 points in item 10. A total score of 10 items was 65 points and was adjusted to 100 points afterward. These 10 items of written tests were tested at pretraining, posttraining after traditional demonstration teaching, and posttraining after VR teaching.

### Statistical Analysis

SPSS for Windows (version 17.0; SPSS Inc) and SAS (version 9.4; SAS Institute) was used for statistical analysis. Quantitative data of “satisfaction of using different learning materials” (reaction level) are presented as mean values with 95% CI. We used nonparametric methods to analyze nonnormal distribution data in this study. Comparisons of pretraining, posttraining after demonstration teaching, and posttraining after VR teaching were scored via written tests and analyzed via Friedman’s test with posthoc analysis, that is, the Dunn-Bonferroni test. The Wilcoxon signed rank test was used to analyze the differences in the “self-assessment questionnaire of participants’ knowledge application” on pretraining and posttraining; the Bonferroni correction was used for multiple comparisons adjustment.

## Results

From August 2021 to January 2022, 31 students from the Department of Medical Laboratory Science and Biotechnology at NCKU in Taiwan used the novel self-developed VR software for 2D VR cognitive testing and 3D VR practical training for BD MAX. In total, 3 of them did not fill out the self-assessment questionnaire for behavior application.

### Kirkpatrick Level I (Reaction): Evaluating Trainees’ Satisfaction

We designed 4 questionnaires to evaluate trainees’ satisfaction and identify their interest in learning. Assessments of the VR teaching material, the design fluency of the VR content, and the improvement in participants’ professional ability were included in the content. The contents of the items are shown in Table 1. The students’ feedback showed that the use of 2D+3D VR teaching materials met students’ needs and enhanced their interest in learning. The item “VR-based teaching materials can increase my interest in learning” exhibited the highest scores in posttraining satisfaction. The results of posttraining satisfaction are shown in Table 1.

**Table 1:** Feedback on the VR^a^ teaching materials and lessons.

Items	Posttraining satisfaction, mean (95% CI)
VR-based assessments to enhance my cognitive learning can improve my learning effectiveness more than traditional written assessments	3.82 (3.43-4.22)
VR-based teaching materials can increase my interest in learning	4.32 (3.99-4.66)
The design fluency of the VR teaching materials was excellent	3.82 (3.41-4.23)
VR teaching materials can meet my learning needs and help me improve my professional ability	3.93 (3.52-4.34)

^a^VR: virtual reality.

### Kirkpatrick Level II (Learning): Determining the Trainees’ Learning Attainment

**Figure 11:**
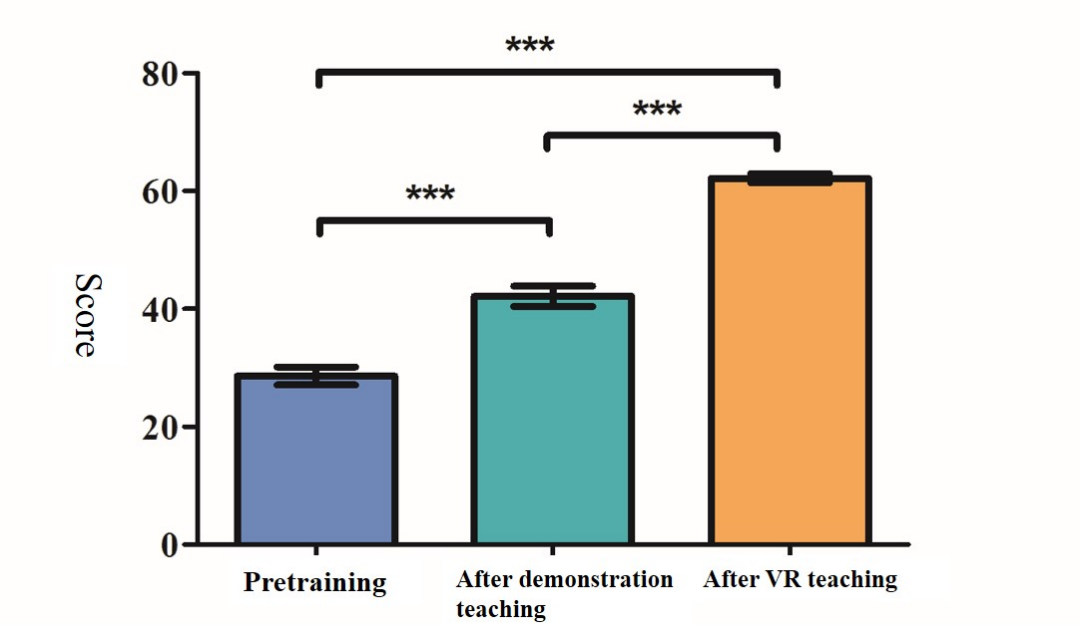
Comparison of written test scores (pretraining, after exposure to traditional demonstration teaching, and after 2D+3D VR teaching) analyzed by Friedman’s test (N=31 out of 100). ***P<.001. VR: virtual reality.

We designed 10 items (Figure 7) to assess students’ background knowledge of HSV or VZV and the operations of BD MAX. There were included in the written tests and 2D VR teaching scenarios. The 3D VR teaching scenarios of the workflow of BD MAX procedures were developed. Written tests assessed students’ knowledge at pretraining, posttraining after exposure to traditional demonstration teaching, and posttraining after exposure to 2D+3D VR teaching. In the analysis of the learning hierarchy, it was found that the average score of 2D+3D VR teaching posttraining was higher than that for traditional demonstration teaching, and the learning effect was significantly better. The data are shown in Figure 11.

### Kirkpatrick Level III (Behavior): Assessing Trainee’s Knowledge Application

**Figure 12:**
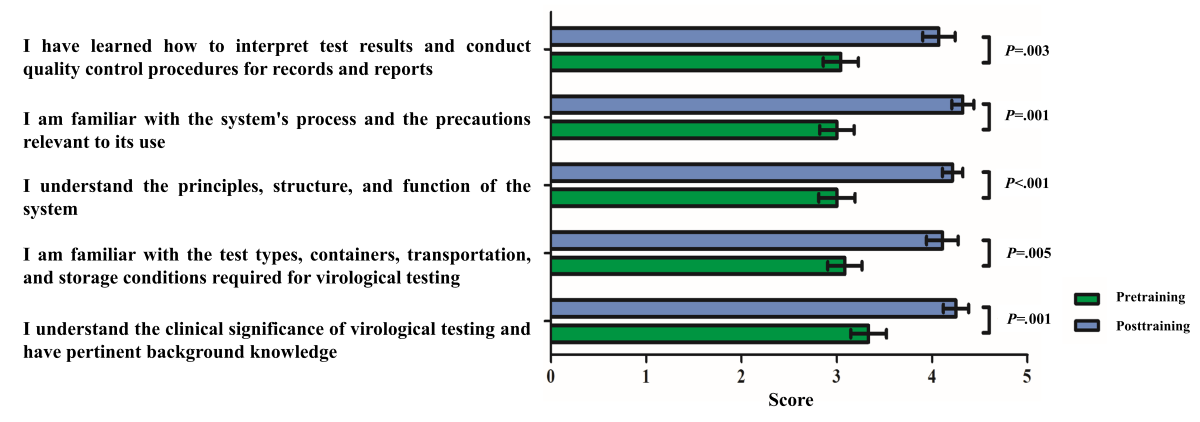
Self-assessment questionnaire of participants’ knowledge application was analyzed by Wilcoxon signed rank test. (N=28, a *P* value was considered significant at the <.01 level after Bonferroni correction [.05/5=.01].).

We designed 5 questionnaires to assess trainees’ knowledge application. The dimensions assessed included interpretation of the results and quality control; familiarity with the BD MAX procedure; understanding the principles, structure, and function of the BD MAX instrument; familiarity with the examination types, containers, transport, and storage conditions; and understanding virological testing’s clinical significance and possessing pertinent background knowledge. The results of participants’ self-assessment pre- and posttraining showed that trainees exposed to VR training to learn about high-level virological testing (using BD MAX) had significantly improved their detailed knowledge posttraining, vis-à-vis pretraining (*P*<.001). The data are shown in Figure 12.

### The 2D Cognitive Software Analyzed the Number of Attempts Before the Correct Answer

**Figure 13:**
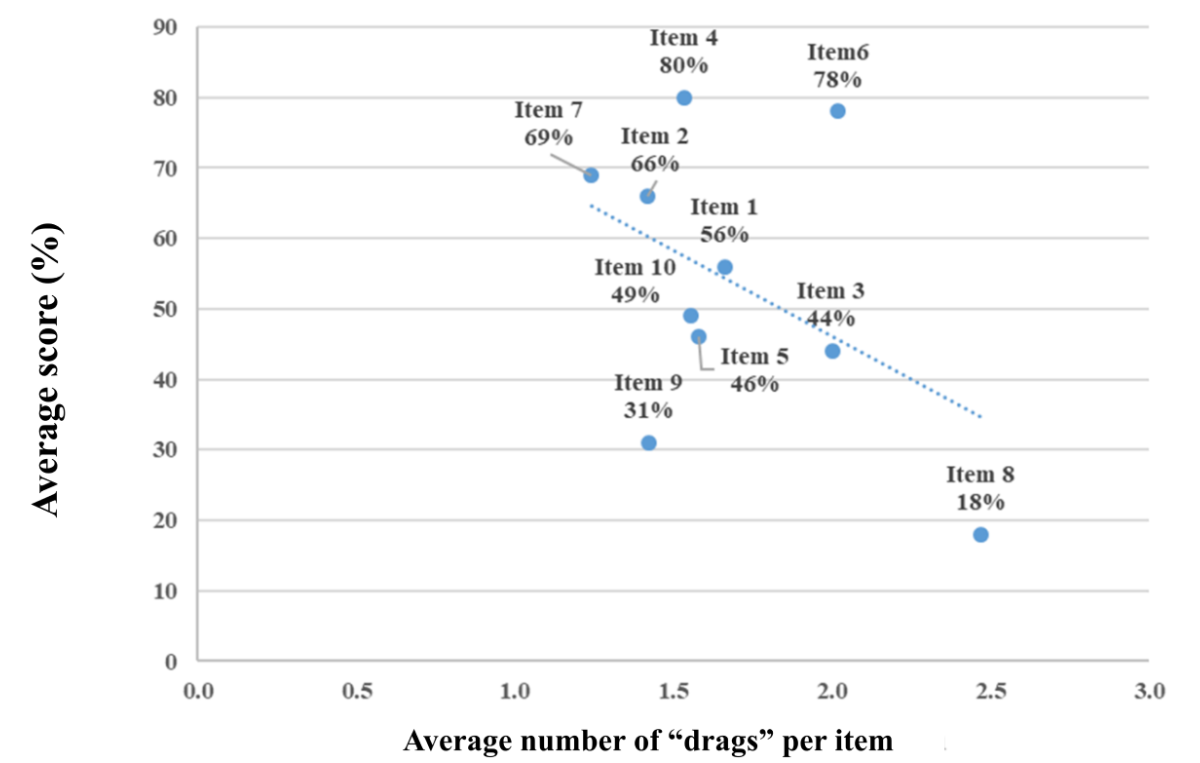
Average number of “drags” vis-à-vis participants’ scores (N=31).

Our team designed a function of the 2D VR teaching software to record the “drags” (ie, attempts) for each item. The drags for each question were recorded and analyzed. The results showed that the higher the interns’ average score, the fewer drags they exhibited. We used this function to assess items’ difficulty level and used it as a reference for adjusting each lesson’s items (Figure 13).

## Discussion

### Main Findings

The use of VR software for teaching has become more popular in clinical medicine. Here, we designed and implemented a VR software for advanced MT training. The participants’ posttraining learning scores increased significantly after being exposed to 2D and 3D VR training. Participants exhibited significantly improved cognition of high-level virological testing posttraining. We analyzed the difficulty of the items and used it as a reference for adjusting them. Participants exposed to VR-based training considered that the VR-based teaching materials met their needs and enhanced their interest in learning.

Developing practical skills is paramount in the field of MT. MT students’ skill-development process begins in the basic experimental courses of natural science during their freshman and sophomore years and continues in the advanced experimental courses related to clinical testing in their junior year as well as the clinical courses in their senior year. MT interns perform more experimental operations than other departments in the medical field. The basic experimental practices performed in school differ significantly from clinical practice courses. In school, the designed laboratory course comprises relatively simple experiments for the students, considering the availability of equipment and resources. However, in the clinical virology practice course of the hospital, additional factors come into play, including the urgency of clinical work, the timeliness and accuracy of reports, the expensive cost of reagents, the complexity of instruments, and the limited number of specimens from patients. Therefore, MT beginners and students rarely have the opportunity to practice in real-world settings.

Due to the rapid development of molecular methodologies, the time required to detect viruses decreased significantly, from several days to 4-6 hours. Current detection procedures can detect multiple pathogens simultaneously, thus, becoming an invaluable tool for the clinical diagnosis of pathogenic infections [9,10,18-21]. However, the test reagents required to detect multiple pathogens simultaneously are expensive (the cost of 1 test is about US $30), and the costs associated with the equipment are also high. The personnel require specialized training to operate the test instrument. Therefore, it is not possible for medical technological interns to practice in hospitals’ clinical virology laboratories. However, schools are also limited by the lack of proper funding, which makes it difficult for them to obtain advanced and expensive instrument for students to practice. Therefore, many intern students are limited to learning from the operation manuals and observing the operation of well-trained medical examiners. Therefore, it is important to use computer technology to develop new teaching methods that allow MT students who do not have access to this specialized technology to receive adequate training.

Mikropoulos et al [22] investigated students’ views on VR learning and found that students generally held positive views regarding the use of VR in teaching while also verifying that VR can capture and maintain students’ attention. Many students expressed that it was exciting to be able to walk in and interact with the 3D VR world. VR simulation allows one to walk inside a molecule to study it in detail or, in the case of a block model, we can acquire a global perspective of a given city block at a distance. This allows the learner to develop a better understanding of the connections between the buildings, open space, and streets. Thus, VR will undoubtedly change the way we look at things and could even allow us to discover things that were previously unimaginable. It could also help people with disabilities break through their physical limitations and engage more actively in learning with their peers.

Of course, not all situations are suitable for the use of VR for education. Pantelidis [23] established the following recommendations regarding the appropriate use of VR: (1) VR would be useful when using real objects is dangerous, impossible, or inconvenient; (2) virtual environments can be taught and trained in the same way as real objects; and (3) interacting with a model is as fun as interacting with the real thing.

Under the conditions of these 3 points, it is recommended to use VR for teaching in clinical virology laboratories. This research project was consistent with all 3 aspects. The BD MAX reagents are expensive, and the number of specimens is limited; thus, it is inconvenient to use a real BD MAX for learning. Other instruments required for PCR molecular detection can be similar to the real object. For interns, working with VR itself is a fun and fresh experience. Therefore, this research project was suitable for simulation using VR. The Centers for Disease Control and Prevention [24] launched a VR teaching video: Biosafety Cabinet Edition in 2021 due to the COVID-19 pandemic.

In addition to the simulated surgical training, VR technology has also been used in baby breathing training, emergency simulation training, dental education, stroke rehabilitation, dental dexterity training, pharmacy teaching, pain management, web-based weight control training, and other clinical applications in the field of medicine and medical care [25-36]. Although all VR devices have similar structures, there are slight differences in hardware and software requirements in different fields as well as different application modes [37-39]. For example, the VR simulation training device for teaching mainly focuses on the use of a 3D or 360-degree environment and network resources, whereas simulations for surgery focus on the interaction between the operator’s instruments and the living anatomy. The environmental design simulations mainly consider the patient’s emotional control and feedback [40-42].

Here, 2D cognitive software and 3D practical VR software of BD MAX lessons were developed. MT interns can use this system to familiarize themselves with the use of virological testing equipment and assess their learning. The results showed that the use of the VR software met the students’ needs and enhanced their interest in learning. In addition, scores from written tests after VR training (posttraining) were significantly higher than after traditional demonstration teaching (posttraining) in the learning level. The behavioral assessment of students pre- and posttraining showed that students exposed to VR training had significantly improved virological knowledge.

### Limitations

The appropriateness of the selection of teaching scripts may have affected the breadth and validity of the use of the teaching software. The fidelity of the 3D elements’ object art and production during VR scenarios requires professional art experts and additional funding support. Due to the limited number of students, this study only has 1 arm and uses a pre- and postcomparison design. Future work will use a randomized 2-arm design to better understand the impact of the technology.

### Conclusions

To the best of our knowledge, this is the first self-developed VR software for high-tech virological testing training. This valuable tool increased MT students’ learning effectiveness. This not only provides beginners and students with the opportunity to operate BD MAX but also saves laboratory and educational costs, thus, enhancing their learning motivation and practical skills. It can also reduce the risk of viral infections particularly during transmitted disease outbreaks. Although VR cannot replace real human interactions, it does have many advantages, such as the freedom to modify, move, and place objects in the virtual world. Its free access is not restricted by time, and it can reduce certain risks. Thus, VR is a valuable teaching tool.

## Appendix

Multimedia Appendix 1 User manual of the self-designed immersive virtual reality training system.

## Abbreviations

BME: biomedical engineering
HSV: herpes simplex virus
MT: medical technology
NCKU: National Cheng Kung University
PCR: polymerase chain reaction
VR: virtual reality

## Data Availability

Data sharing is not included in our original institutional review board so we will not share data in this submission.

## References

[1] Delahoy MJ, Ujamaa D, Whitaker M, O'Halloran A, Anglin O, Burns E, Cummings C, Holstein R, Kambhampati AK, Milucky J, Patel K, Pham H, Taylor CA, Chai SJ, Reingold A, Alden NB, Kawasaki B, Meek J, Yousey-Hindes K, Anderson EJ, Openo KP, Teno K, Weigel A, Kim S, Leegwater L, Bye E, Como-Sabetti K, Ropp S, Rudin D, Muse A, Spina N, Bennett NM, Popham K, Billing LM, Shiltz E, Sutton M, Thomas A, Schaffner W, Talbot HK, Crossland MT, McCaffrey K, Hall AJ, Fry AM, McMorrow M, Reed C, Garg S, Havers FP, COVID-NET Surveillance Team (2021). Hospitalizations associated with COVID-19 among children and adolescents—COVID-NET, 14 states, March 1, 2020–August 14, 2021. MMWR Morb Mortal Wkly Rep.

[2] Liu WJ, Xiao H, Dai L, Liu D, Chen J, Qi X, Bi Y, Shi Y, Gao GF, Liu Y (2021). Avian influenza A (H7N9) virus: from low pathogenic to highly pathogenic. Front Med.

[3] Stein RT, Bont LJ, Zar H, Polack FP, Park C, Claxton A, Borok G, Butylkova Y, Wegzyn C (2017). Respiratory syncytial virus hospitalization and mortality: systematic review and meta-analysis. Pediatr Pulmonol.

[4] Tsai HP, Kuo PH, Liu CC, Wang JR (2001). Respiratory viral infections among pediatric inpatients and outpatients in Taiwan from 1997 to 1999. J Clin Microbiol.

[5] Lee DH, Zuckerman RA, AST Infectious Diseases Community of Practice (2019). Herpes simplex virus infections in solid organ transplantation: guidelines from the American Society of Transplantation Infectious Diseases Community of Practice. Clin Transplant.

[6] Stern A, Papanicolaou GA (2019). CMV prevention and treatment in transplantation: what's new in 2019. Curr Infect Dis Rep.

[7] Tsai HP, Yeh CS, Lin IT, Ko WC, Wang JR (2020). Increasing cytomegalovirus detection rate from respiratory tract specimens by a new laboratory-developed automated molecular diagnostic test. Microorganisms.

[8] Levin MJ, Weinberg A, Schmid DS (2016). Herpes simplex virus and varicella-zoster virus. Microbiol Spectr.

[9] Pillet S, Verhoeven PO, Epercieux A, Bourlet T, Pozzetto B (2015). Development and validation of a laboratory-developed multiplex real-time PCR assay on the BD Max system for detection of Herpes simplex virus and Varicella-Zoster virus DNA in various clinical specimens. J Clin Microbiol.

[10] Tansarli GS, Chapin KC (2020). Diagnostic test accuracy of the BioFire® FilmArray® meningitis/encephalitis panel: a systematic review and meta-analysis. Clin Microbiol Infect.

[11] Bric JD, Lumbard DC, Frelich MJ, Gould JC (2016). Current state of virtual reality simulation in robotic surgery training: a review. Surg Endosc.

[12] Zahiri M, Nelson CA, Oleynikov D, Siu KC (2018). Evaluation of augmented reality feedback in surgical training environment. Surg Innov.

[13] Al-Azri H, Ratnapalan S (2014). Problem-based learning in continuing medical education: review of randomized controlled trials. Can Fam Physician.

[14] Jin J, Bridges SM (2014). Educational technologies in problem-based learning in health sciences education: a systematic review. J Med Internet Res.

[15] Jones-Bonofiglio KD, Willett T, Ng S (2018). An evaluation of flipped e-learning experiences. Med Teach.

[16] Rucker SY, Ozdogan Z, Al Achkar M (2017). Flipped classroom model for learning evidence-based medicine. Adv Med Educ Pract.

[17] Tang F, Chen C, Zhu Y, Zuo C, Zhong Y, Wang N, Zhou L, Zou Y, Liang D (2017). Comparison between flipped classroom and lecture-based classroom in ophthalmology clerkship. Med Educ Online.

[18] Huang HS, Tsai CL, Chang J, Hsu TC, Lin S, Lee CC (2018). Multiplex PCR system for the rapid diagnosis of respiratory virus infection: systematic review and meta-analysis. Clin Microbiol Infect.

[19] Koehler JW, Douglas CE, Minogue TD (2018). A highly multiplexed broad pathogen detection assay for infectious disease diagnostics. PLoS Negl Trop Dis.

[20] Poritz MA, Blaschke AJ, Byington CL, Meyers L, Nilsson K, Jones DE, Thatcher SA, Robbins T, Lingenfelter B, Amiott E, Herbener A, Daly J, Dobrowolski SF, Teng DH, Ririe KM (2011). FilmArray, an automated nested multiplex PCR system for multi-pathogen detection: development and application to respiratory tract infection. PLoS One.

[21] Stockmann C, Pavia AT, Graham B, Vaughn M, Crisp R, Poritz MA, Thatcher S, Korgenski EK, Barney T, Daly J, Rogatcheva M (2017). Detection of 23 gastrointestinal pathogens among children who present with diarrhea. J Pediatric Infect Dis Soc.

[22] Mikropoulos TA, Chalkidis A, Katsikis A, Emvalotis A (1998). Students' attitudes towards educational virtual environments. Educ Inf Technol (Dordr).

[23] Pantelidis VS (2009). Reasons to use virtual reality in education and training courses and a model to determine when to use virtual reality. Themes Sci Technol Educ.

[24] LabTrainingVR: biosafety cabinet edition, virtual reality laboratory training. Centers for Disease Control and Prevention.

[25] Ezenwa BN, Umoren R, Fajolu IB, Hippe DS, Bucher S, Purkayastha S, Okwako F, Esamai F, Feltner JB, Olawuyi O, Mmboga A, Nafula MC, Paton C, Ezeaka VC (2022). Using mobile virtual reality simulation to prepare for in-person helping babies breathe training: secondary analysis of a randomized controlled trial (the eHBB/mHBS trial). JMIR Med Educ.

[26] Lerner D, Mohr S, Schild J, Göring M, Luiz T (2020). An immersive multi-user virtual reality for emergency simulation training: usability study. JMIR Serious Games.

[27] Li Y, Ye H, Ye F, Liu Y, Lv L, Zhang P, Zhang X, Zhou Y (2021). The current situation and future prospects of simulators in dental education. J Med Internet Res.

[28] Kiper P, Szczudlik A, Agostini M, Opara J, Nowobilski R, Ventura L, Tonin P, Turolla A (2018). Virtual reality for upper limb rehabilitation in subacute and chronic stroke: a randomized controlled trial. Arch Phys Med Rehabil.

[29] Aramaki AL, Sampaio RF, Reis ACS, Cavalcanti A, Dutra FCMSE (2019). Virtual reality in the rehabilitation of patients with stroke: an integrative review. Arq Neuropsiquiatr.

[30] Ben-Gal G, Weiss EI, Gafni N, Ziv A (2013). Testing manual dexterity using a virtual reality simulator: reliability and validity. Eur J Dent Educ.

[31] Coyne L, Merritt TA, Parmentier BL, Sharpton RA, Takemoto JK (2019). The past, present, and future of virtual reality in pharmacy education. Am J Pharm Educ.

[32] Li A, Montaño Z, Chen VJ, Gold JI (2011). Virtual reality and pain management: current trends and future directions. Pain Manag.

[33] Malloy KM, Milling LS (2010). The effectiveness of virtual reality distraction for pain reduction: a systematic review. Clin Psychol Rev.

[34] Piskorz J, Czub M (2018). Effectiveness of a virtual reality intervention to minimize pediatric stress and pain intensity during venipuncture. J Spec Pediatr Nurs.

[35] Thomas JG, Spitalnick JS, Hadley W, Bond DS, Wing RR (2015). Development of and feedback on a fully automated virtual reality system for online training in weight management skills. J Diabetes Sci Technol.

[36] Li L, Yu F, Shi D, Shi J, Tian Z, Yang J, Wang X, Jiang Q (2017). Application of virtual reality technology in clinical medicine. Am J Transl Res.

[37] Shahzad A (2019). Culminate coverage for sensor network through bodacious-instance mechanism. J Wirel Commun Netw.

[38] Ashraf S, Alfandi O, Ahmad A, Khattak AM, Hayat B, Kim KH, Ullah A (2020). Bodacious-instance coverage mechanism for wireless sensor network. Wirel Commun Mob Comput.

[39] Ashraf S, Ahmed T, Saleem S (2021). NRSM: node redeployment shrewd mechanism for wireless sensor network. Iran J Comput Sci.

[40] Liu K, Fang B, Wu Y, Li Y, Jin J, Tan L, Zhang S (2013). Anatomical education and surgical simulation based on the Chinese visible human: a three-dimensional virtual model of the larynx region. Anat Sci Int.

[41] Vaughan N, Dubey VN, Wainwright TW, Middleton RG (2016). A review of virtual reality based training simulators for orthopaedic surgery. Med Eng Phys.

[42] Patel D, Hawkins J, Chehab LZ, Martin-Tuite P, Feler J, Tan A, Alpers BS, Pink S, Wang J, Freise J, Kim P, Peabody C, Bowditch J, Williams ER, Sammann A (2020). Developing virtual reality trauma training experiences using 360-degree video: tutorial. J Med Internet Res.

