# Supplementary material for: Novel Software for High-level Virological Testing: Self-Designed Immersive Virtual Reality Training Approach
Source: J Med Internet Res. 2023 Jun 21;25:e44538. doi: 10.2196/44538 (PMC10337313; doi:10.2196/44538)

**Multimedia Appendix 1.** User manual of the self-designed immersive virtual reality training system.


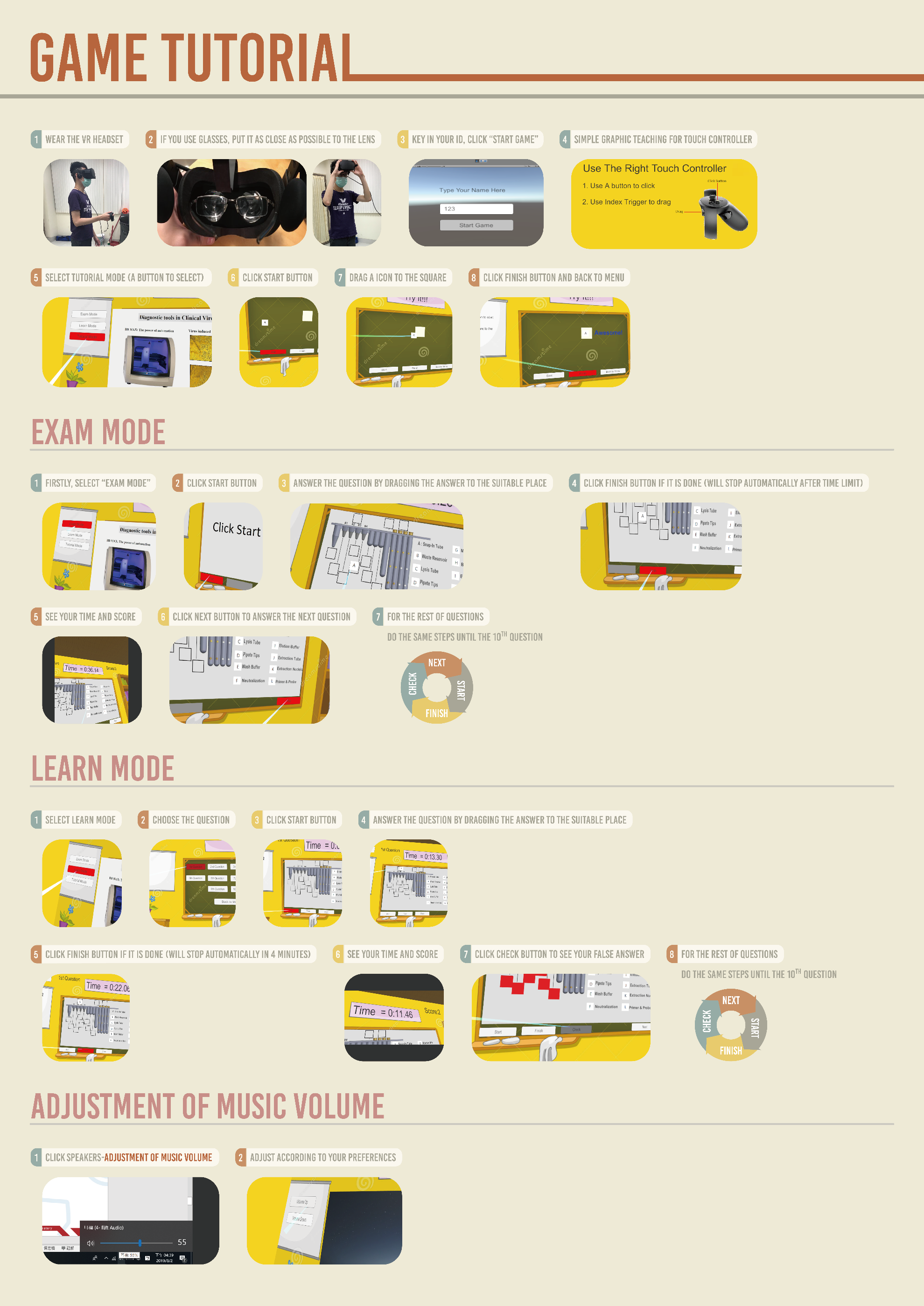

Supplement: Multimedia Appendix 1 [file jmir_v25i1e44538_app1.docx]
